# Automated grading of enlarged perivascular spaces in clinical imaging data of an acute stroke cohort using an interpretable, 3D deep learning framework

**DOI:** 10.1038/s41598-021-04287-4

**Published:** 2022-01-17

**Authors:** Brady J. Williamson, Vivek Khandwala, David Wang, Thomas Maloney, Heidi Sucharew, Paul Horn, Mary Haverbusch, Kathleen Alwell, Shantala Gangatirkar, Abdelkader Mahammedi, Lily L. Wang, Thomas Tomsick, Mary Gaskill-Shipley, Rebecca Cornelius, Pooja Khatri, Brett Kissela, Achala Vagal

**Affiliations:** 1 Department of Radiology, University of Cincinnati, 234 Goodman Street, Cincinnati, OH 45267 USA; 2 Division of Biostatistics and Epidemiology, Cincinnati Children’s Hospital Medical Center, Cincinnati, OH USA; 3 Department of Pediatrics, University of Cincinnati College of Medicine, Cincinnati, OH USA; 4 Department of Neurology, Cincinnati Children’s Hospital Medical Center, Cincinnati, OH USA; 5 Department of Neurology and Rehabilitation Medicine, University of Cincinnati College of Medicine, Cincinnati, OH USA; 6 I-MED Radiology Network, Melbourne, VIC Australia

**Keywords:** Magnetic resonance imaging, Translational research

## Abstract

Enlarged perivascular spaces (EPVS), specifically in stroke patients, has been shown to strongly correlate with other measures of small vessel disease and cognitive impairment at 1 year follow-up. Typical grading of EPVS is often challenging and time consuming and is usually based on a subjective visual rating scale. The purpose of the current study was to develop an interpretable, 3D neural network for grading enlarged perivascular spaces (EPVS) severity at the level of the basal ganglia using clinical-grade imaging in a heterogenous acute stroke cohort, in the context of total cerebral small vessel disease (CSVD) burden. T2-weighted images from a retrospective cohort of 262 acute stroke patients, collected in 2015 from 5 regional medical centers, were used for analyses. Patients were given a label of 0 for none-to-mild EPVS (< 10) and 1 for moderate-to-severe EPVS (≥ 10). A three-dimensional residual network of 152 layers (3D-ResNet-152) was created to predict EPVS severity and 3D gradient class activation mapping (3DGradCAM) was used for visual interpretation of results. Our model achieved an accuracy 0.897 and area-under-the-curve of 0.879 on a hold-out test set of 15% of the total cohort (n = 39). 3DGradCAM showed areas of focus that were in physiologically valid locations, including other prevalent areas for EPVS. These maps also suggested that distribution of class activation values is indicative of the confidence in the model’s decision. Potential clinical implications of our results include: (1) support for feasibility of automated of EPVS scoring using clinical-grade neuroimaging data, potentially alleviating rater subjectivity and improving confidence of visual rating scales, and (2) demonstration that explainable models are critical for clinical translation.

## Introduction

Enlarged Perivascular Spaces (EPVS) is a key, but understudied, component in assessing cerebral small vessel disease burden after stroke (CSVD). While EPVS has been associated with worse cognition, depression, and neurodegenerative disorders, its full prognostic significance is unknown^1^. A key limitation is the use of visual rating scales used to grade EPVS severity with poor inter-rater reliability, limiting internal and external validity of findings^2^. Development of accurate, reliable, and *interpretable* automated EPVS scoring based on clinical data could circumvent this issue, aiding research on mechanisms of EPVS, improving knowledge of clinical significance, and assisting large studies assessing EPVS as a biomarker for clinical outcomes^2^. Interpretability is vital to translational aspects of deep learning models, including model verification, enhancing trust in model predictions, and fixing errors leading to misclassifications^3,4^.

The clinical significance of the current study is two-fold. First, EPVS rating, specifically in the basal ganglia, has been shown to correlate strongly with other measures of CSVD, cognitive impairment at 1 year after ischemic stroke, and stroke risk factors in hemorrhagic stroke cohorts^5,6^. Additionally, previous studies have found that EPVS rating scores in the basal ganglia are commonly not normally distributed and have found that a meaningful categorization for logistic regression to be 10 punctate EPVS, where < 10 is considered none-to-mild and ≥ 10 is considered moderate-to-severe^7^. Using this dichotomy, EPVS was shown to be associated with EPVS in the centrum semiovale and atrophy.

The second point of clinical significance of the current study is the utility of an automatic stratification tool (low-risk vs high-risk for CSVD) for clinical grade imaging. Because speed of acquisition is the primary goal of an acute clinical scan, the slice thickness acquired is usually much thicker than research-grade imaging. For the present study, the average slice thickness of the scans used was 4 mm, whereas EPVS are usually defined as punctate fluid-containing spaces < 3 mm when measured perpendicular to the vessel^8^. While the axial resolution of the scan is high enough to capture these, the limited slice thickness means that some EPVS may not be able to be resolved clearly, making it much harder to delineate single points in the 5-point EPVS rating scale. Having a tool that can automatically, and quickly, identify patients with moderate-to-severe EPVS would greatly facilitate studies of CSVD since this delineation has been shown to correlate with the other markers of total CSVD burden.

Previous attempts to segment or classify EPVS have limited generalizability due primarily to data quality and cohort selection. Prior studies have used ultra-high filed MRI (7 T), which is rarely available for clinical use, with high-resolution scans that require a very long scan time. Additionally, the cohorts used in these studies have been volunteers, greatly reducing the chance of excessive image noise/artifact^9–13^. The purpose of the current study was to develop an interpretable and clinically generalizable 3D neural network for grading EPVS severity using clinical-grade imaging in an acute stroke cohort. Studies attempting to grade EPVS typically use research-grade images that do not generalize well to standard-of-care protocols. We hypothesized that we could achieve an accuracy of at least 76%, based on previous studies of EPVS scoring inter-rater reliability^1^ and that network visualizations would be physiologically plausible.

The strength of the current study compared to previous studies is that results will be maximally generalizable because: (1) EPVS rating was assessed by 5 central readers, accounting for biases that may arise from reader tendencies, (2) images were collected at multiple sites, accounting for site-specific variation in data collection, (3) the images used were clinical-grade images that may feasibly be collected at any site capable of MRI, and (4) the study cohort included all types of strokes, including imaging-negative trans-ischemic attack (TIA) patients, so the results should generalize to various patient populations. This study was designed, and manuscript prepared according to the checklist for artificial intelligence in medical imaging (CLAIM)^14^.

## Results

### Data

There were 143 patients with none-to-mild EPVS and 119 patients with moderate-to-severe EPVS. Demographic information can be found in Supplementary Table 1. Notably, this cohort included patients with various types of strokes, including those who had imaging negative transient ischemic attacks. This suggests results may be generalizable to non-stroke patients as well.

### Model performance

**Figure 1:**
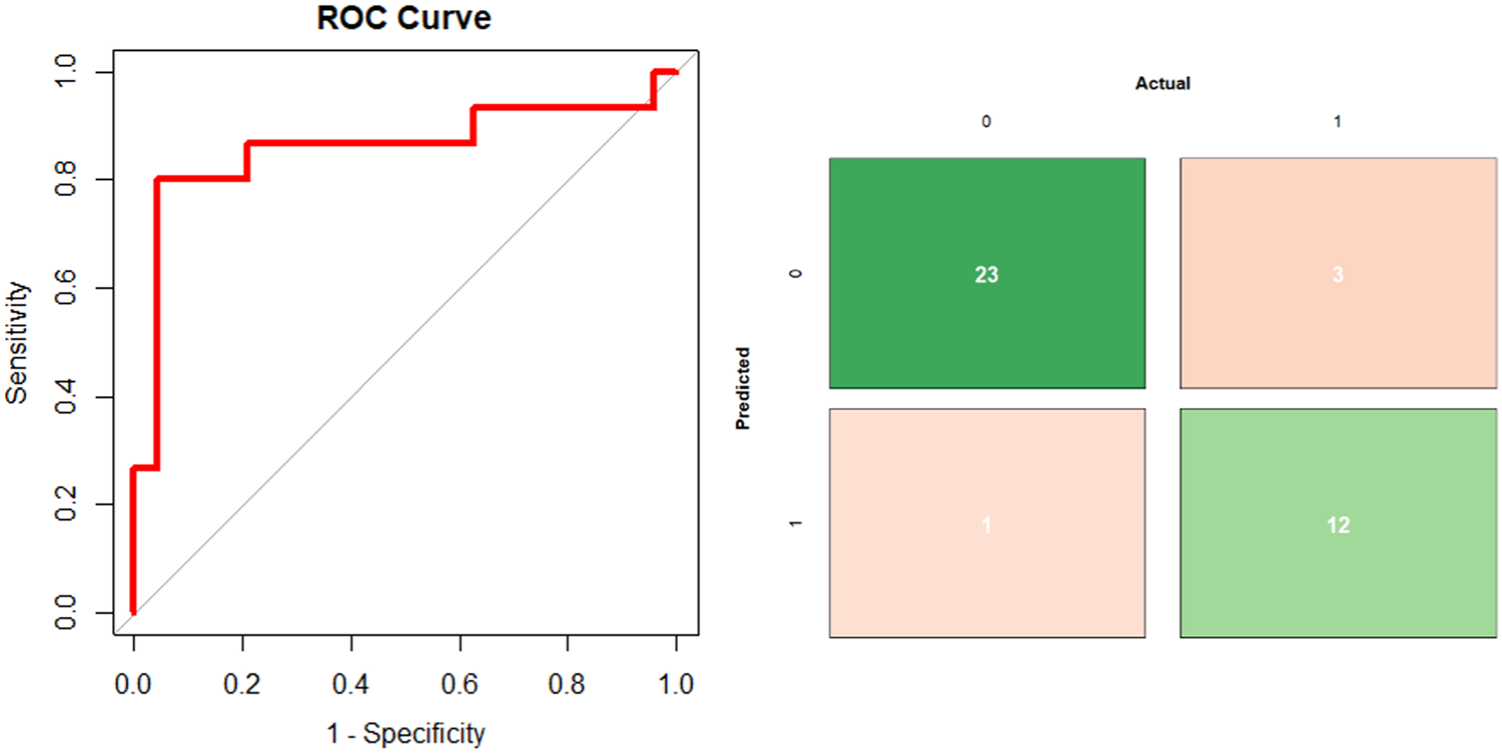
Assessment of model performance. The model achieved an Accuracy/AUC of 0.897/0.879 on the test set (left panel). In the confusion matrix (right panel), 0 indicates none-to-mild EPVS and 1 indicates moderate-to-severe EPVS. Out of 39 samples, there were 3 false positives and 1 false negative.

Our final model, ResNet-152, achieved an accuracy/AUC of 0.802/0.834 on the training set, 0.768/0.847 on the validation set, and 0.897(95% CI = [0.758, 0.971])/0.879 on the test set (Fig. 1, left panel) for detection of none-to-mild versus moderate-to-severe EPVS. The positive class is defined as moderate-to-severe EPVS and the negative class is defined as none-to-mild EPVS. On the held-out test set, specificity was 0.96, sensitivity was 0.80, and F1 was 0.86. The model had a positive predictive value of 92.31% and a negative predictive value of 88.46%. Accuracy was significantly higher than the NIR (NIR = 0.617; *p* < 0.001). There were 3 false negatives and 1 false positive (Fig. 1, right panel). In the false positive case, the model picked up on remote infarcts that resemble EPVS. Mean CLEVER score for the test set was 5.76, indicating that the model was substantially robust to noise. Supplementary table 2 shows the comparison of validation loss and accuracy for the three models that were tested (ResNet-50, ResNet-101, ResNet-152). For the best model from this process, dropout was tuned to maximize validation accuracy. The best network based on fivefold cross-validation accuracy was ResNet-152 with 40% dropout (Supplementary Table 2).

**Figure 2:**
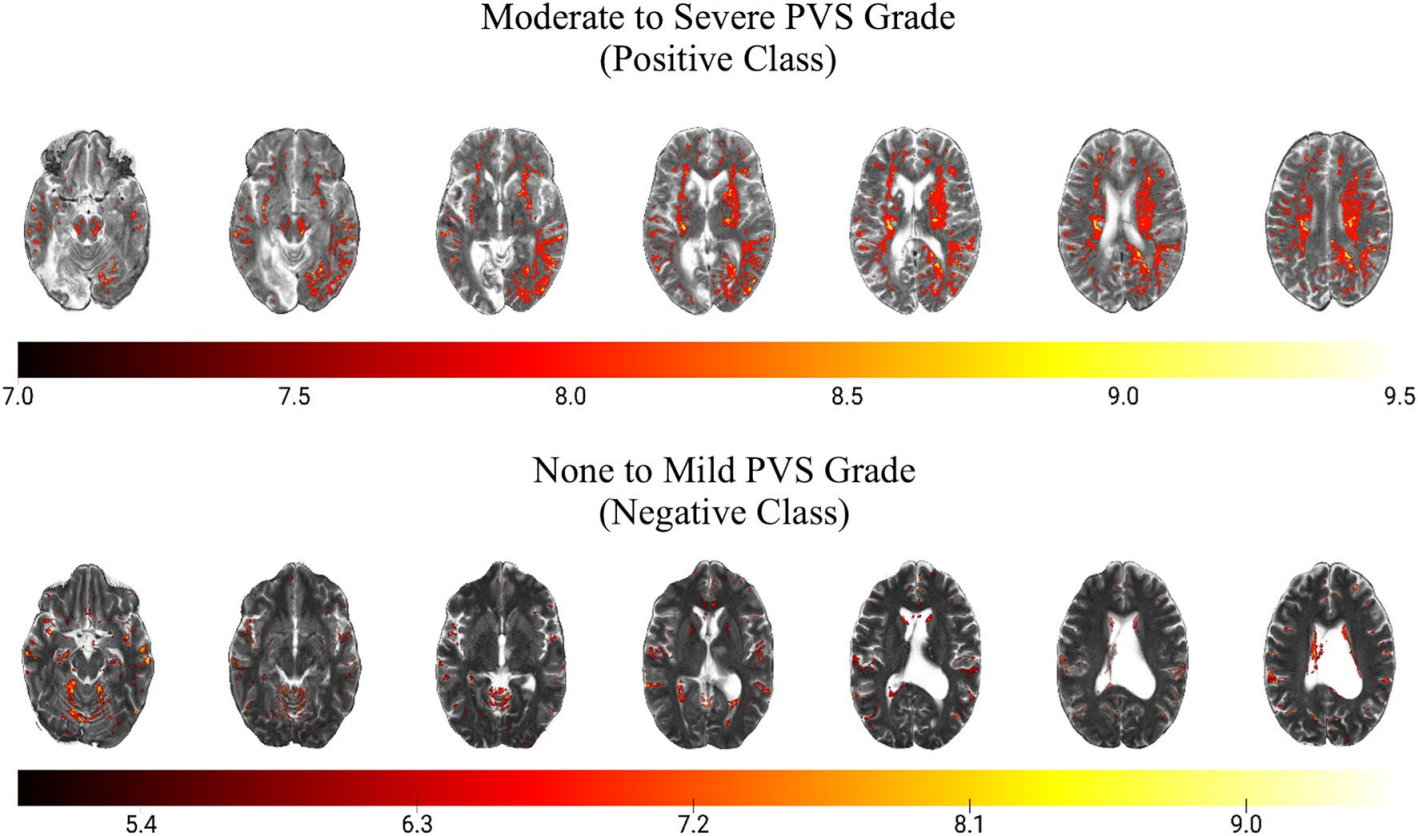
3D gradient class activation maps (3DGradCAM) showing prototypical activations for examples in each classification (positive versus negative). Positive examples showed high activation in several relevant midline regions, including midbrain, basal ganglia, and centrum semiovale (top panel). Negative examples had fewer activations in the high activation range (> 7) and smaller activations localized in non-relevant (random) areas (bottom panel).

**Figure 3:**
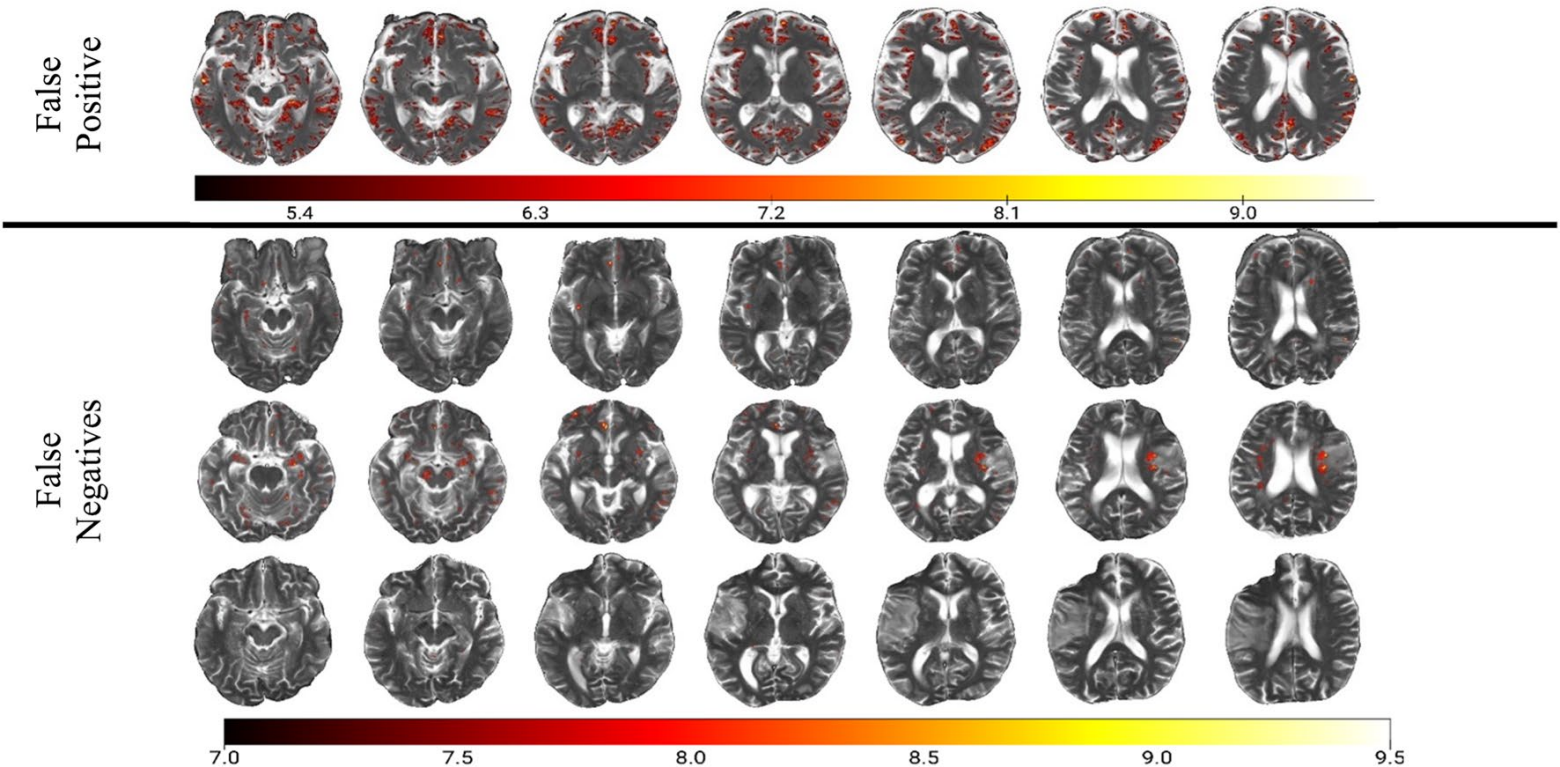
Analysis of misclassified patients. In the false positive case, the network seems to have picked up on remote infarcts that often resemble EPVS. In the false negative cases, there were less activations in the high range (> 7), i.e. activations were more homogenous.

3DGradCAM revealed that midline regions, including midbrain, basal ganglia, and centrum semiovale, with high-valued activations (> 7) were indicative of severe EPVS (Fig. 2, top panel). In none-to-mild examples, fewer regions had high activations, and these lower-valued activations localized in non-relevant hyperintense tissue (Fig. 2, bottom panel). Misidentified examples suggested the distribution of class activations was the primary cause of error. In the false positive case, more tissue was resolved in the high range than in the true negative cases (Fig. 3, top row). For false negatives, less areas were resolved in the high range than in the true positive cases (Fig. 3, bottom 3 rows).

## Discussion

We demonstrate that an explainable deep learning model can feasibly classify patients with moderate-to-severe EPVS using only standard-of-care T2-weighted imaging. The model performed as hypothesized, and activation maps were consistent with expected anatomy. While only EPVS scores at the level of the basal ganglia were used, the model focused on both basal ganglia and other relevant regions, indicating possible correlative abnormalities. Activations were high in much of the white matter, but the highest activations (most yellow regions in Fig. 3, top row) were in regions most associated with EPVS (centrum semiovale, basal ganglia, midbrain). While there have been attempts to quantify and segment EPVS and the neural network architecture we employed is not unique, no prior studies have used the approach of the current study because: (1) prior studies have used high-resolution imaging (0.5 × 0.5 × 0.8 mm) and complex preprocessing pipelines that cannot generalize to clinical imaging, (2) used patches of tissue centered around regions-of-interest instead of the whole brain and/or, (3) used multimodal imaging data^2,15–17^. While these studies have provided finer details on the nature of EPVS, high resolution imaging is simply not possible in many acute settings. The key contribution of this study is that the current model could be used for efficient patient risk stratification, in the context of total CSVD burden, using only a clinical T2-w image.

As previously mentioned, prior studies involving automated pipelines for EPVS classification/segmentation are not necessarily clinically generalizable since the methods used may not be feasible in all clinical situations. For example, two recent studies adequately segmented EPVS, with Dice score ranging from 0.62 to 0.66 for unimodal imaging, and up to 0.77 for multimodal imaging. However, the clinical utility, including for clinical trials, is severely limited as these studies were performed on data collected on 7 T scanners, which are widely not available, with high-resolution protocols that take more than 10 min per acquisition, on average, leading to unfeasible scan times^9–13^.

Removal of the “black box” with explainable AI models is important for clinical translation. 3DGradCAM maps indicating activation distribution plays an important role in model classification. By providing the model prediction, along with a saliency map and statistics of the activation distribution, a radiologist would be better able to interpret the model output. These activation maps allow understanding of which parts of the image are being used by the model. A moderate-to-severe EPVS prediction with a physiologically viable saliency map and negatively skewed activation distribution would give the radiologist more confidence in the decision. One explanation for false positive findings, apparent in the analysis of misclassified images, is the presence of hyperintense lacunar infarcts that resemble and often coexist with EPVS. This insight can be used to inform ongoing training of the model and improve its clinical applicability.

Our study has important limitations including the size of the test set, which may skew model evaluation. However, since this set was not observed until the final evaluation, the performance is still substantial. Model performance will continue to be evaluated as new data are collected. Another limitation is data were not stratified by more variables. Future analyses will determine whether this has a significant impact. Another potential limitation is the image preprocessing necessary for adequate model performance. The ground truth was derived from the original images. The purpose of the preprocessing, especially registration, was to limit the search space of the network, reducing the need for more data. Preprocessing was minimal and takes only ~ 3 min per subject, so the utility for quick stratification is not lost with its inclusion. But future studies will consider this limitation and aim to have adequate sample sizes so less preprocessing is necessary for good model generalization.

It is also notable that the test set accuracy/AUC were slightly higher than the validation set. This is likely due to more noise/variability in the validation set compared to the test set. With a bigger sample size, this difference should normalize so that values are comparable. Finally, this study included patients only from an acute stroke cohort. Future studies will include other populations, such as typical aging, to validate findings.

In conclusion, we show that explainable models are feasible and provide information that increase confidence in the model’s decision, allowing for use in a clinical setting. While the strict dichotomization of EPVS into the two groups used here is not necessarily clinically meaningful, the insights gained from model interpretation potentially are. Additionally, the ability to quickly randomize patients into those with and without significant EPVS severity, based on previous CSVD burden literature. This would facilitate large-scale clinical trials of EPVS and total CSVD burden, which are much needed. Future studies will use larger datasets to explore methods to improve upon the current results and use probabilistic modeling to quantify model confidence.

## Methods

### Study design

A retrospective cohort from an ongoing population-based acute stroke study (APRISE; R01 NINDS NS103824-01) was used. A convenience subset of 348 patients was selected based on: (1) presence of an axial T2-weighted image and (2) grading of EPVS severity score at the level of the basal ganglia. T2-w images were chosen as they are the most used modality to grade EPVS and were the most prevalent scan in each dataset. Due to the acute nature of the scans, there is typically very limited time to collect data, therefore making higher resolution scans unfeasible. Additionally, many of the scans had significant motion artifact. After excluding scans of poor quality, 262 unique patients remained. Scans were removed if there was enough noise in the image to obscure judgement of EPVS rating in the basal ganglia, as determined by visual inspection. This may be able to be automated in future studies but is most reliably done manually. Since this is a proof-of-concept study, we wanted to include only scans that were of adequate quality, determined by image clarity. A flowchart of the patients that were included is provided in supplementary Fig. 1. This study was approved by the local Institutional Review Board of the University of Cincinnati and consent waived due to the retrospective nature of the study. All study activities were carried out in accordance with the Declaration of Helsinki and all data analyzed was anonymized.

### Data preprocessing

T2-weighted scans were collected from 5 different sites, including academic and community hospitals, all consistent in sequence type and axial resolution. Data were stored and de-identified using AMBRA (http://ambrahealth.com). Scans were rigidly aligned to a base image, chosen as a high-quality example that had a median number of slices (range = 24–36, median = 32), resampled, and skull removed. Alignment and skull stripping were performed with AFNI^18^. Next, images were scaled, windowed, and cropped to include only the middle 16 slices. We chose to include only the middle 16 slices because, upon visualization of all participants, this range completely captured the basal ganglia for all scans while excluding extraneous slices. After visualizing all datasets, the optimal intensity contrast was determined to be between 82 and 90% quantiles. The process for determining this threshold range was: (1) several lower thresholds were tested until EPVS were thresholded out for any single participant and (2) several upper thresholds were tested until EPVS were clearly distinguishable from adjacent intensity values for every participant. These preprocessing steps narrowed the search space for the final model and were performed in R 4.0.3^19^.

### Ground truth

Ground truth was determined by 5 expert neuroradiologists, based on a previously published EPVS rating scale^1^. All readers were initially assigned the same set of 30 training cases to assess inter-rater reliability. After training to resolve discrepancies, a further 15 cases were assigned. Inter-rater reliability for EPVS scoring was 0.64 (Gwet’s AC2 statistic for ordinal score). While this reliability score is in the good range, it is important to keep in mind that this rating came from 5 neuroradiologists who subsequently discussed the cases in which there was a major disagreement (2 or more points on the rating scale). Therefore, agreement is likely greater than this initial assessment and there feasibly exists a latent “truth” for each of these ratings, if averaged across all readers. However, future studies will seek to address the determination of an optimal ground truth for this task.

### Data partitions

Data were split into training and test sets by an 85%-15% split, stratified by EPVS severity (0 for < 10, 1 for ≥ 10). Binarizing the data was necessary primarily due to data quality, the amount of data available, and the relevance to the prediction of CSVD. Since the spatial resolution of the images are not optimal, it is difficult to classify each category of the 5-point rating scale used to grade EPVS. This binarization is consistent with the definition of EPVS in the calculation of total CSVD^20^. The training set was split into training and validation sets by a 75%-25% split, stratified by the same criteria, resulting in training, validation, and test sets of 167, 56, and 39, respectively. Data also varied by central reader, study site, and stroke subtype, but the limited sample size did not allow for stratification by these variables.

### Model

**Figure 4:**
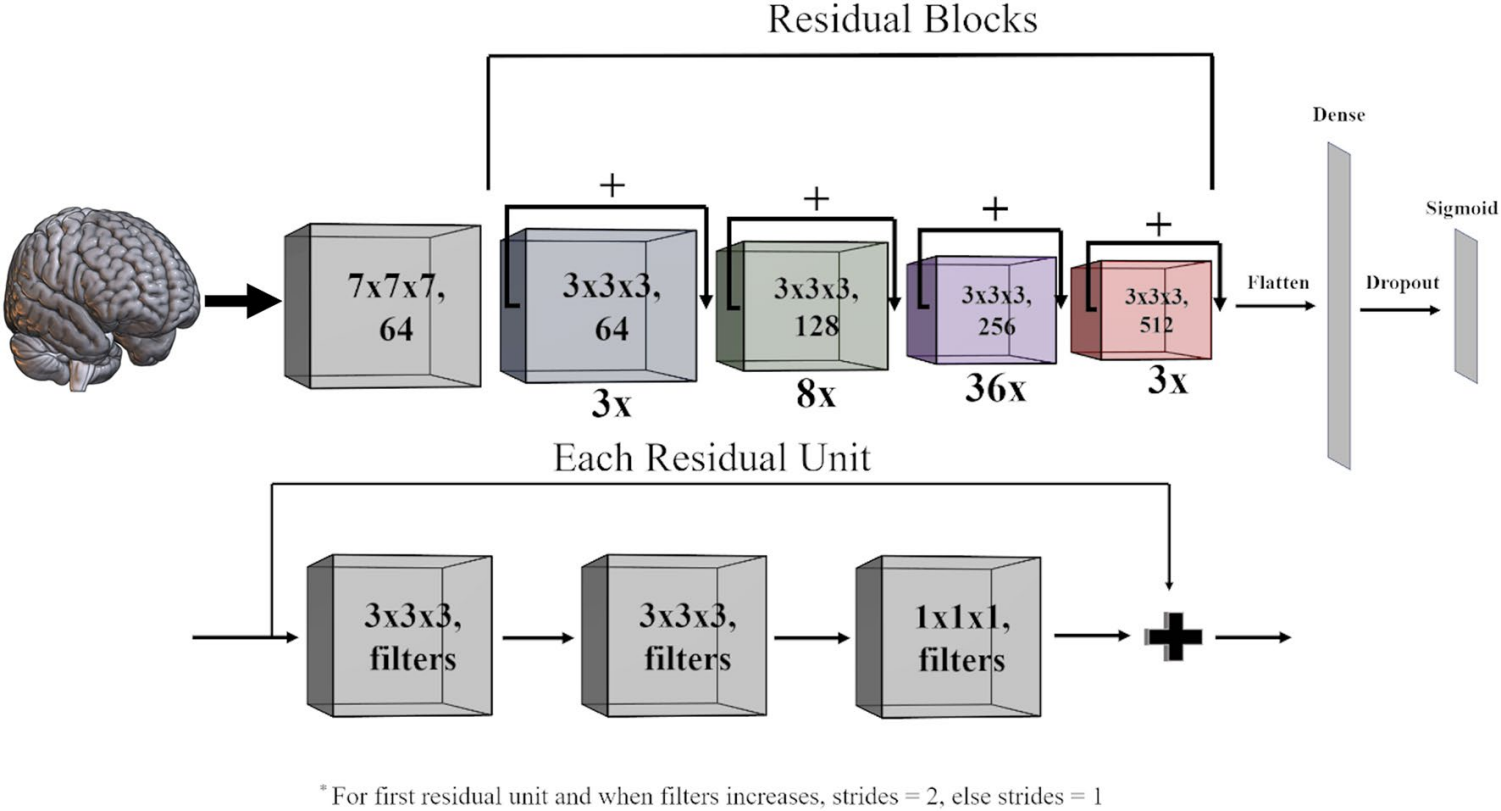
Schematic of the 3D-ResNet-152 that was used for this analysis. Each image was first passed through a 3D convolutional layer (7 × 7 × 7, 64 filters) with ReLu activation and batch normalization, followed by a series of 50 residual units, each with 3 convolutional layers (bottom panel). This output was relayed to a fully-connected dense layer with one output and sigmoid activation (top panel).

A 3D-152-layer Residual Network (ResNet) was used for classification^21^. Input (512 × 512 × 16 × 1) was fed into an initial 3D-convolutional layer (64 filters, kernel size = 7 × 7 × 7, strides = 2), followed by batch normalization, rectified linear unit (ReLu) activation, and max pooling (pool size = 3, stride = 2). Then came a series of 50 residual units (3 with 64 filters, 8 with 128 filters, 36 with 256 filters, and 3 with 512 filters). Strides were set to 2 for the first residual unit and when filter size increased, and 1 otherwise. Each residual unit consisted of three 3D-convolutional layers, the first two with kernel size of 3 × 3 × 3 and the last with kernel size of 1 × 1 × 1. At the end of each residual unit, the input was passed through the last layer and added to the output. Output from the last residual unit was fed into a global average layer, flattened, and passed to a fully-connected dense layer with 1 output and sigmoid activation (Fig. 4). ReLu activation and batch normalization were implemented after each convolutional layer. Before the final layer, dropout of 40% was used to decrease overfitting. Glorot uniform initialization was used for all layers. TensorFlow in R was used for modeling.

### Training

An Adam optimizer (learning rate = 0.001) was used on binary cross-entropy during training^22^. Overfitting was reduced by model checkpointing, which monitored validation area-under-the-curve (AUC) and reducing the learning rate by a factor of 0.1 when validation loss plateaued for 10 epochs. Batch size was equal to 20. A total of 3 models were tested (ResNet-50, ResNet-101, and ResNet-152) and dropout was tuned on the final model. The final model and dropout rate used for this model were selected using stratified cross-validation. Results from this procedure can be found in supplementary Table 2. Final model selection was based on results that best balanced training and validation AUC.

### Evaluation

Accuracy and AUC were used for model evaluation. The ‘no-information rate’ (NIR) was used to determine whether model accuracy was statistically significant, and a binomial test was used to compute 95% confidence intervals. Average Cross-Lipschitz Extreme Value for Network Robustness (CLEVER) score was calculated for the ℓ2-norm set to assess robustness^23^. 3D gradient class activation mapping (3DGradCAM) was used to produce normalized saliency maps (range = 0–10)^24^.

## Supplementary Information

Supplementary Table S1.

Supplementary Table S2.

Supplementary Figure S1.

## Data Availability

Code can be found at: https://github.com/willi3by/PVSNet. Model weights can be provided upon request.
