# Supplementary material for: Automated grading of enlarged perivascular spaces in clinical imaging data of an acute stroke cohort using an interpretable, 3D deep learning framework
Source: Sci Rep. 2022 Jan 17;12:788. doi: 10.1038/s41598-021-04287-4 (PMC8764081; doi:10.1038/s41598-021-04287-4)
Supplement: Supplementary file 1 — Supplementary Table S1. [file 41598_2021_4287_MOESM1_ESM.docx]

Supplementary Table 1. Patient characteristics.

|  | Training Set  (N = 223) | Test Set  (N = 39) |
| --- | --- | --- |
| Age in years, mean (SD), median [IQR] | 72.2 (14.6)  74 [63-85] | 74.2 (11.1)  74 [66-83] |
| Sex – Female | 109 (49%) | 25 (64%) |
| Race |  |  |
| White | 183 (82%) | 32 (82%) |
| Black | 37 (17%) | 6 (15%) |
| Asian | 3 (1%) | 1 (3%) |
| NIH Stroke Scale, mean (SD), median [IQR] | 6.0 (8.7)  2 [1-7] | 6.7 (9.2)  2 [1-9] |
| Hypertension | 198 (89%) | 30 (77%) |
| Diabetes | 87 (39%) | 10 (26%) |
| Smoking |  |  |
| Current | 30 (14%) | 3 (8%) |
| Ever | 79 (36%) | 15 (38%) |
| Never | 108 (50%) | 21 (54%) |
| Type of stroke |  |  |
| Transient Ischemic Attack | 44 (20%) | 9 (23%) |
| Infarct | 154 (69%) | 24 (62%) |
| Subarachnoid Hemorrhage | 3 (1%) | 2 (5%) |
| Intracerebral Hemorrhage | 22 (10%) | 4 (10%) |

SD = standard deviation; IQR = interquartile range.

Data shown as number (percentage) unless noted otherwise.
