# Supplementary material for: Automated grading of enlarged perivascular spaces in clinical imaging data of an acute stroke cohort using an interpretable, 3D deep learning framework
Source: Sci Rep. 2022 Jan 17;12:788. doi: 10.1038/s41598-021-04287-4 (PMC8764081; doi:10.1038/s41598-021-04287-4)
Supplement: Supplementary file 2 — Supplementary Table S2. [file 41598_2021_4287_MOESM2_ESM.docx]

*Table 1.* Results of 5-fold Nested Cross-Validation for each model architecture and dropout percentage. Each network was trained with 50 epochs and batch size of 12.

| **Model** | **Dropout** | **Validation Accuracy %**  **(mean +/- sd)** | **Validation loss**  **(mean +/- sd)** |
| --- | --- | --- | --- |
| ResNet-50 | 0% | 64.93 +/- 5.7 | 7.95 +/- 1.29 |
| ResNet-101 | 0% | 60.93 +/- 6.1 | 8.54 +/- 0.90 |
| ResNet-152 | 0% | 68.99 +/- 6.2 | 7.95 +/- 1.28 |
| ResNet-152 | 20% | 65.45 +/- 2.8 | 8.74 +/- 0.59 |
| ResNet-152 | 30% | 67.66 +/- 5.6 | 8.74 +/- 0.59 |
| ResNet-152 | 40% | 69.90 +/- 8.2 | 8.19 +/- 1.20 |
| ResNet-152 | 50% | 69.42 +/- 5.2 | 8.34 +/- 0.75 |
| ResNet-152 | 60% | 67.27 +/- 4.7 | 8.74 +/- 0.59 |
