# Supplementary figures and images for: Automated grading of enlarged perivascular spaces in clinical imaging data of an acute stroke cohort using an interpretable, 3D deep learning framework
Source: Sci Rep. 2022 Jan 17;12:788. doi: 10.1038/s41598-021-04287-4 (PMC8764081; doi:10.1038/s41598-021-04287-4)

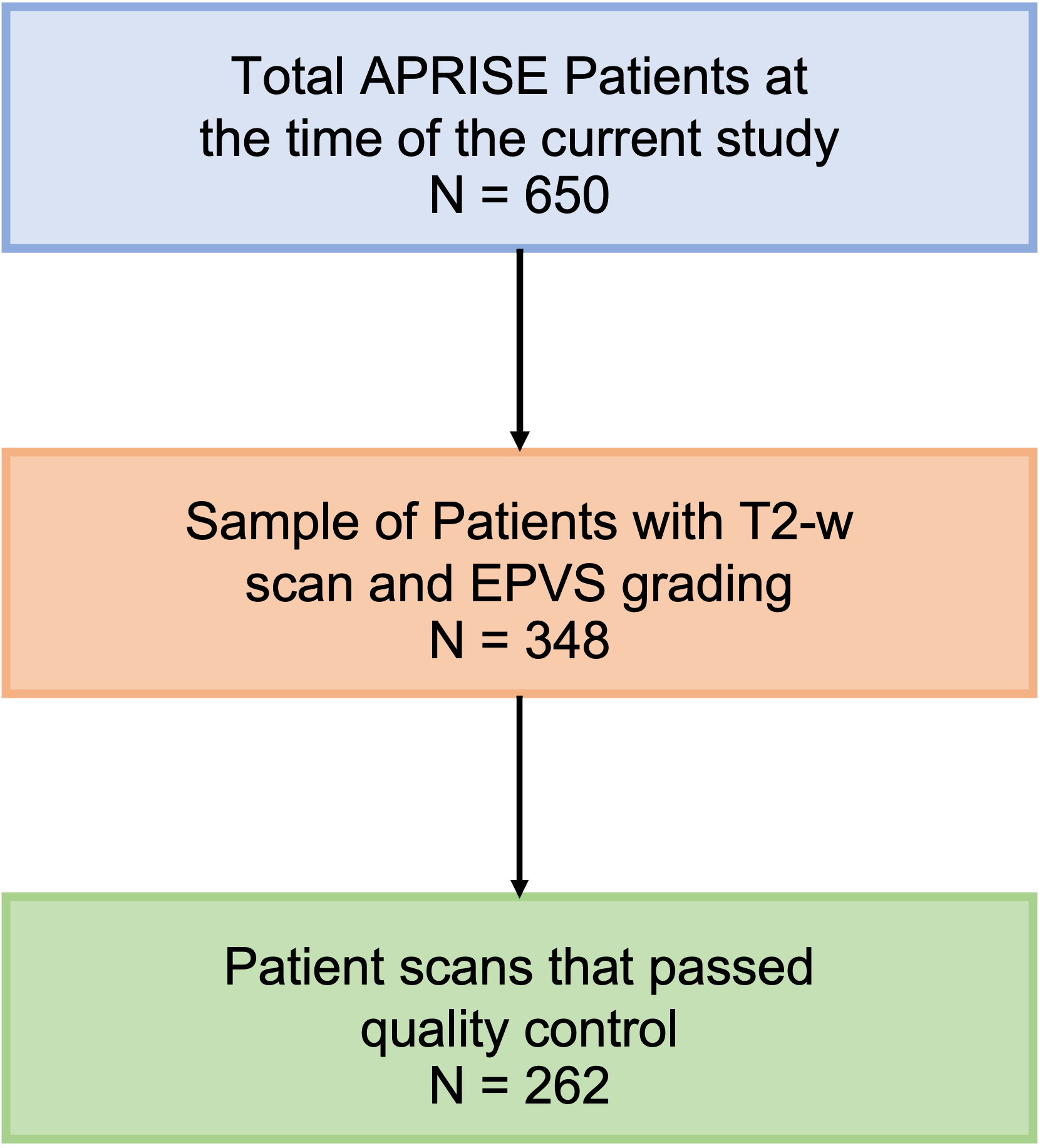

Supplement: Supplementary file 3 — Supplementary Figure S1. [file 41598_2021_4287_MOESM3_ESM.tiff]
